# Metschnikowia maris comb. nov., a large-spored yeast species endemic to Serra do Mar Atlantic Rainforest biome, Sao Paulo State, Brazil

**DOI:** 10.1099/ijsem.0.006891

**Published:** 2025-08-20

**Authors:** Ana Raquel de Oliveira Santos, Giovana Reis de Ávila, Olavo Henrique Petrucci Della Torre, Sérgio Luiz Pompéia, Maria da Graça Stupiello Andrietta, Carlos A. Rosa, Marc-André Lachance

**Affiliations:** 1 Centro de Coleções Taxonômicas da UFMG, ICB, Universidade Federal de Minas Gerais, Belo Horizonte, MG, Brazil; 2 Departamento de Microbiologia, ICB, Universidade Federal de Minas Gerais, Belo Horizonte, MG, Brazil; 3 Instituto de Pesquisas da Biodiversidade (IPBio), Reserva Betary, Iporanga, SP, Brazil; 4 Centro Pluridisciplinar de Pesquisas Químicas e Biológicas, Universidade Estadual de Campinas, Estrada PL, Betel, Paulinia, SP, Brazil; 5 Department of Biology, University of Western Ontario, London, Ontario, Canada

**Keywords:** ascomycete, *Metschnikowia maris*, new combination, Serra do Mar, yeast

## Abstract

Two yeast isolates from passion flowers were sampled in the southern part of the Serra do Mar Atlantic Rainforest in Sao Paulo State, Brazil. Barcode sequencing and mating experiments showed them to be representatives of *Metschnikowia matae* var. *maris*, thus originally named due to the availability of only a single isolate and uncertainties regarding reproductive isolation. The two new isolates being of the complementary mating type to the previously known strain, intravarietal crosses were performed. They yielded a preponderance of two-spored asci, unlike crosses with *M. matae* var. *matae*, which led to largely sterile asci. We therefore elevate the variety *maris* to the rank of species, with the name *Metschnikowia maris* comb. nov. The holotype is UFMG-CM-Y397^T^ (*MAT*α). Strain UFMG-CM-Y7613^A^ (*MAT***a**) is designated as allotype. The new combination is registered as MB 859665.

## Introduction

The large-spored *Metschnikowia* clade consists of haplontic, heterothallic species whose needle-shaped ascospores well exceed 50 µm in length. Most have been recovered from species of *Conotelus*, a nitidulid beetle that is frequently found in the flowers of morning glories and other plant species. Isolations may also be from corollas or nectar of flowers visited by other insects, including Nitidulidae. The range is global, but most species are endemic to various regions of mostly tropical biomes. To date, these regions include East and South Africa; Australia; Hawaii; North, Central and South America; and, most recently, India [1,2]. Brazil shares some species with other regions but is the only known source for *Metschnikowia continentalis*, *Metschnikowia cerradonensis*, *Metschnikowia amazonensis* and *Metschnikowia matae*, the latter with two varieties [3].

*M. matae* var. *matae* was described from 20 isolates recovered in 3 localities of the Atlantic Forest ecoregion (Mata Atlantica) in the states of Rio de Janeiro, Minas Gerais and Bahia [4]. In contrast, the variety *maris* was known from the more southern Sao Paulo State, but only from a single isolate, which was part of the motivation for reporting it as a variety instead of a full-fledged species. Other elements of the circumscription included a borderline divergence in barcode sequences and a reduced fertility in intervarietal crosses. More specifically, matings among compatible mating types of the variety *matae* gave rise to a high proportion of two-spored asci, whereas mating involving the single isolate of the variety *maris* with compatible *matae* counterparts resulted instead in mostly empty or single-spored asci. Lachance and Bowles [5] showed that the latter result correlates with ascus sterility. The average nucleotide identity (OrthoANI) determined for the genomes of a pair of strains of the variety *matae* was 97.3, which is well above the threshold typical of conspecifics, whereas intervarietal values of 94.0 and 94.1 argued somewhat in favour of distinct species [6]. The missing clue remained whether matings among strains of the variety *maris* would be fertile. The alternative possibility was that the unique strain assigned to the separate variety was simply deficient in the complex process that leads to the formation of fertile asci.

If yeast systematics is to be a predictive science, and not just ‘stamp collecting’ (*secundum* Ernest Rutherford [7], p. 9), one should be able to integrate the information gathered at the time of collection to generate hypotheses that can later be either verified or falsified. The distribution of *M. matae* suggested a north-south gradient with the variety *matae* in more northerly latitudes and the variety *maris* more to the south, which allowed one to predict that intensified sampling in similar southern habitats would generate additional strains of the variety *maris*, including those of the opposite (*MAT***a**) mating type. Indeed, a recent foray conducted in a more southerly location of the Serra do Mar Atlantic Forest biome in Sao Paulo State yielded two isolates that provided good grounds to elevate the variety *maris* to the rank of species.

## Methods

Collections were conducted in the Betary Reserve, Iporanga, Sao Paulo State, in April 2024. This is a site of Atlantic Rain Forest located at the Serra do Mar biome (24.59 S 48.63 W). Twenty passion flowers were collected, placed in sterile plastic bags and processed on the same day of collection. The basal portion of each flower, containing the nectary, was aseptically excised and transferred to sterile tubes containing 20 ml of enrichment medium [50% glucose (w/w), 0.5% yeast extract and 0.01% chloramphenicol] and incubated at 25 °C for 10 days. Upon visible yeast growth, the cultures were homogenized by shaking, and decimal dilutions were prepared for each sample. Aliquots of 100 µl from appropriate dilutions were plated onto YM (yeast-malt) agar (1% glucose, 0.3% yeast extract, 0.3% malt extract, 0.5% peptone, 2% agar and 0.01% chloramphenicol) and incubated at 25 °C for 10 days. Distinct yeast morphotypes were subsequently purified by repeated streaking on YM agar and preserved at −80 °C. Growth characteristics were determined by replica plating following the methods recommended by Kurtzman *et al*. [8]. Mating and ascus formation were observed in pairwise mixtures on Yeast Carbon Base agar with 0.01% yeast extract over 3-day incubation at room temperature (25±3 °C).

Identification was performed by sequencing the internal transcribed spacer (ITS) region, including the 5.8S rRNA gene, as well as the D1/D2 domains of the LSU rRNA gene, following Santos *et al*. [4]. Sequencing was performed at the Fundação Oswaldo Cruz (FIOCRUZ), Belo Horizonte, Brazil. The amplified DNA was cleaned and sequenced with an ABI 3130 Genetic Analyzer automated sequencing system using BigDye v3.1 and POP7 polymer. Sequences were edited and aligned using the MAFFT [9] plugin of the Geneious Prime platform. Sequences were compared with those in the GenBank database using the Basic Local Alignment Search Tool (blast; https://blast.ncbi.nlm.nih.gov). A haplotype network based on aligned ITS sequences was generated with the program TCS [10], using all changes, including gaps, in calculating the number of steps. The parsimony test was overridden by setting the connection limit to 50 steps.

## Results and discussion

A total of 29 yeast isolates were obtained, as summarized in Table 1. The predominant species recovered was *Kodamaea ohmeri*, followed by representatives of the genus *Starmerella*, which accounted for 6 of the 17 identified species. Notably, *Hanseniaspora opuntiae* and *Metschnikowia dekortorum* were isolated from the same passionflower as strain UFMG-CM-Y7613. Similarly, *Priceomyces* sp. and *Starmerella* sp. were recovered from the flower that yielded strain UFMG-CM-Y7614. The low incidence of large-spored *Metschnikowia* species and the range of other yeast species encountered suggest that the flowers were visited by a wide variety of insects, where *Conotelus* sp. is a minor component.

**Fig. 1.**
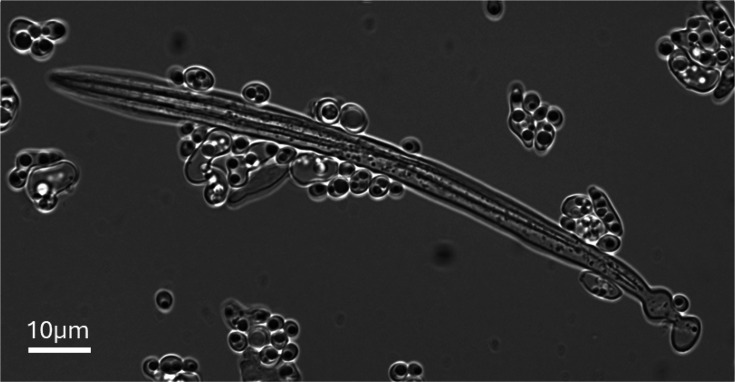
Mature ascus of *M. maris* comb. nov. arising from a cross between strains UFMG-CM-Y397 (*MAT*α) and UFMG-CM-Y7613 (*MAT**a***), showing the formation of two ascospores. Phase contrast.

**Fig. 2.**
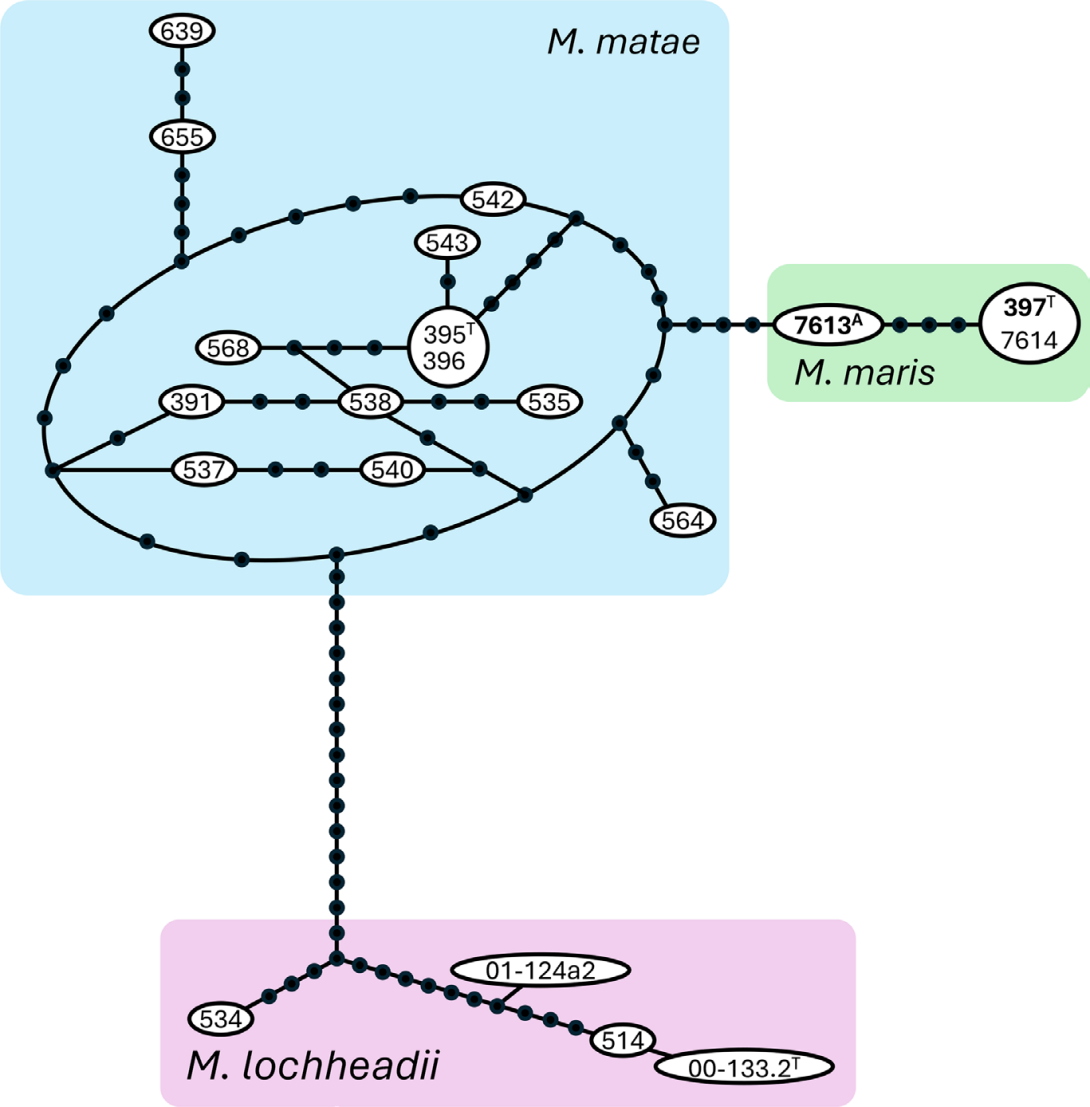
Haplotype network of the ITS rDNA regions of *M. maris* comb. nov. and close relatives. Each circle represents one change (substitution or gap). The numbers are abbreviated from those in the Collection of Microorganisms of the Universidade Federal de Minas Gerais, Belo Horizonte, MG, Brazil (3–4 digits), and the University of Western Ontario, Department of Biology Yeast Culture Collection (6–7 digits).

Two isolates were identified by sequencing the ITS region and D1/D2 domains as belonging to *Metschnikowia matae* var. *maris*. Identification was confirmed by mixing suitable isolates in every possible pair together and with ex-types of the two varieties of *M. matae* (UFMG CM-Y395^T^ and Y397^T^). The new isolates, UFMG-CM-Y7613^A^ and UFMG-CM-Y7614, had mating type **a**. The new isolates (Y7613^A^ and Y7614) formed abundant two-spored asci when mixed with the type of variety *maris* (Fig. 1), as observed also in the positive control cross involving the type and allotype of the variety *matae*. Intervarietal crosses led to mostly empty asci that often were smaller and less well developed. A few one- or two-spored asci were also present. The phylogenetic placement of *M. matae* has been amply documented by phylogenomics [11,12] and needs no elaboration here, except to say that the species forms a subclade with other large-spored *Metschnikowia* species found predominantly in Central and South America. On a finer scale, Santos *et al*. [4] presented a haplotype network based on the rDNA region spanning the ITS and the D1/D2 LSU rRNA gene, which showed the clearly distinct status of *M. matae* from *Metschnikowia lochheadii* and the slightly divergent status of the variety *maris*. We repeated this analysis with the sequences of the new isolates, using ITS sequences only, as the two varieties differed by only a single indel in the D1/D2 region. As seen in Fig. 2, the new isolate UFMG-CM-Y7613^A^ (GenBank PV809773) differs at three positions from the type of the variety *maris* and nine positions from the nearest isolates of the variety *matae* in the network. Hence, a discontinuity exists between the varieties, although it may not on its own make a convincing case to treat them as separate species. A key element in the present case is the formation of mostly two-spored asci in crosses among isolates assigned to *Metschnikowia maris* comb. nov. and mostly sterile asci in crosses between them and strains of *M. matae*. The distinct status of the species is also complemented at the whole-genome level by the OrthoANI value of 94%. Values below 95% are generally observed for yeasts that are deemed to represent different species based on reproductive isolation in the large-spored *Metschnikowia* species [6], and a similar threshold seems to apply to species of other yeast genera [13]. Both studies, however, indicate that values from 94 to 96% represent a zone of uncertainty and should be interpreted with circumspection, in context.

**Table 1.** Frequency of occurrence of yeast species in *Passiflora* sp. flowers collected in Serra do Mar Atlantic Forest biome

Species	Flower (*n*=20)
*H. opuntiae*	1
*Hanseniaspora uvarum*	2
*Hyphopichia burtonii*	1
*K. ohmeri*	5
*Kurtzmaniella quercitrusa*	1
*M. dekortorum*	1
*M. maris* comb. nov.	2
*Metschnikowia* sp. (identical to ST-57 – GenBank accession DQ400374)	1
*Meyerozyma carpophila*	1
*Priceomyces* sp. (identical to UFMG-CM-Y6328 – GenBank MG737686)	1
*Starmerella apicola*	2
*Starmerella bacillaris*	1
*Starmerella etchellsii*	3
*Starmerella litoralis*	1
*Starmerella meliponinorum*	3
*Starmerella* sp. (identical to LESF 1454 – GenBank ON493995)	2
*Torulaspora pretoriensis*	1
Total isolates	29

## *Metschnikowia maris* (A.R.O. Santos, C.A. Rosa & Lachance) comb. nov. A.R.O. Santos, C.A. Rosa & Lachance

Basionym : *Metschnikowia matae* var. *maris* A.R.O. Santos, C.A. Rosa and Lachance, Antonie van Leeuwenhoek 108 (3): 761 (2015).

Etymology: ma’ris. L. gen. n. *maris*, of the sea, in reference to the habitat in the Atlantic Rainforest biome.

The species description is the same as given for *Metschnikowia matae* var. *maris* by Santos *et al*. [4]. The holotype is strain UFMG-CMY397^T^ (mat**α**) recovered from an unidentified flower in Serra do Mar State Park, São Paulo, Brazil, previously deposited as the holotype of the variety *maris*. It is preserved in a metabolically inactive form in the Microbial Culture Collection of the University of Minas Gerais, Belo Horizonte, Minas Gerais, Brazil. Isotypes have been deposited (CBS 13985, NRRL Y-63737). The MycoBank number is MB 859665. Strain UFMG-CM-Y7613 (*MAT***a**) is an allotype. The ITS-D1/D2 LSU rRNA gene region sequence has been deposited in GenBank under accession number PV809773.

## Abbreviations

ITS: internal transcribed spacer
